# Health benefits of new symbiotic “NAR”

**DOI:** 10.5195/cajgh.2013.114

**Published:** 2014-01-24

**Authors:** Saule Saduakhasova, Almagul Kushugulova, Samat Kozhakhmetov, Gulnara Shakhabayeva, Adil Supiyev, Zhanagul Khasenbekova, Indira Tynybayeva, Talgat Nurgozhin, Zhaxybay Zhumadilov

**Affiliations:** Center for Life Sciences, Nazarbayev University, Astana, Kazakhstan

**Keywords:** synbiotics, immune modulation, antioxidant activity

## Abstract

**Introduction:**

The immune-modulatory effects of synbiotics and their ability to reduce free radical levels may be useful for functional food that is able to be active throughout whole period of colonization of the gastrointestinal tract.

The aim of the present study was to investigate the immune-modulatory and antioxidant effects of the synbiotic product “NАR,” a probiotic beverage.

**Methods:**

The presence of IL-2, IL-4, IL-6, IL-8, IL-10, αTNF, γIFN, Ig A, Ig M, and Ig E was studied in vitro using a solid immunosorbent analysis. The total antioxidant activities of superoxide dismutase and glutathione reductase were determined by a spectrophotometry using the Sigma-Aldrich sets.

**Results:**

Studies of the immune-modulatory properties of the synbiotic product NAR showed 1.7 fold increase of γINF levels (*p*<0.01) in blood after consumption of the synbiotic product “NAR” in comparison to control values, whereas the concentrations of IL-4 and Ig E decreased 2.0 times (treatment: 9.3; control: 18.7; *p*<0.01) and 1.3 times (*p*<0.1), respectively. The consumption of the synbiotic product “NAR” caused an increase in the proportion of γINF/IL 4 (treatment: 15.4; control: 4.4; *p*<0.01), which indicates a reduction in functional activity of Th2-type lymphocytes in comparison with the function of Th1 cells.

Our study showed a high level of the total antioxidant activity of the synbiotic product (67.4 mmol/ml). The antioxidant activity of the intact cells of consortium (15.3 mM/ml), which was the basis for the preparation of the symbiotic product, is several times lower than the activity observed in the symbiotic samples.

Expression of SOD is one of the mechanisms of antioxidant stress radicals inactivation by bacteria. The analysis identified a superoxide dismutase activity of synbiotic product (1.42 U/mg protein). A glutathione reductase activity of the synbiotic product was elevated (0.06 U/ml).

**Conclusion:**

The majority of the inflammatory mediators found in the blood after the consumption of symbiotic product NAR were inflammatory mediators that activate a cellular component of the resistance. Moreover, the symbiotic product has a high antioxidant activity.

